# High polymorphism detected by massively parallel sequencing of autosomal STRs using old blood samples from a Chinese Han population

**DOI:** 10.1038/s41598-019-55282-9

**Published:** 2019-12-12

**Authors:** Wenshen Dai, Yajiao Pan, Xiaochen Sun, Riga Wu, Luo Li, Dongming Yang

**Affiliations:** 1 Beijing Institute of Biomedicine, Beijing, 100091 P.R. China; 2 IPE Biotechnology Co., Ltd., Beijing, 100176 P.R. China; 3 Faculty of Forensic Medicine, Zhongshan School of Medicine, Sun Yat-sen University, Guangzhou, 510080 P.R. China; 4 Guangdong Province Translational Forensic Medicine Engineering Technology Research Center, Sun Yat-sen University, Guangzhou, 510080 P.R. China

**Keywords:** Next-generation sequencing, Next-generation sequencing, Next-generation sequencing

## Abstract

The development of massively parallel sequencing (MPS) has quickly changed forensic short tandem repeat (STR) genotyping. By providing detailed sequence information, MPS technology may be used as an alternative or additional method to overcome the limitations of capillary electrophoresis-based STR profiling. Most current NGS processes are labour-intensive with regard to library preparation and require high-quality DNA template. In this study, a 16-plex STR typing system (SeqType^®^R16) was used to achieve direct library preparation without DNA extraction and adaptor ligation. The efficiency of this system was tested in 601 individuals, including 593 old blood samples from the Chinese Han population and eight positive controls. It took approximately 4 hours for library preparation, including blood direct multiplex PCR (1.5 hours), mixing of the product (15 minutes), single tube purification (2 hours) and quantification (15 minutes). The results showed that MPS presented a broader allele range and higher discrimination power. Except for FGA and D19S433, the allele number almost doubled or more than doubled at all complex STR loci and simple STR loci, including D13S317, D16S539, D5S818, and D7S820. The range of discrimination power increased from 0.8008–0.9572 to 0.8401–0.9753, and the culminated matching probability decreased from 1.7 × 10^−15^ to 1.1 × 10^−17^.

## Introduction

Since the 1990s, the application of short tandem repeats (STRs) in forensic science has become a standard genetic marker for individual discrimination and paternity identification^1^. The STR profile can be obtained by fluorescently labelled multiplex amplification and capillary electrophoresis separation, which is dependent on length variations among individuals^1,2^. Commercial kits for STR loci detection have been developed and updated to 6-dye fluorescence labelling kits, such as the GlobalFiler^TM^ Express Kit, which detects more STR loci simultaneously and prevents overlap of amplification products^3^.

Massively parallel sequencing (MPS) technology, also referred to as next-generation sequencing (NGS), offers a new, high-throughput research method for biological sciences^4^. Over the years, an increasing number of researchers have begun using NGS technology for forensic applications because it can generate thousands of sequences in a single reaction. The most important aspect of NGS is that it can be used to sequence STR loci and provide accurate composition information, including the length and repeat structure of the product^5^. Compared with traditional capillary electrophoresis-based STR (CE-STR), NGS technology is not limited by the number of fluorescent dyes or the number of loci with overlapping size ranges. Therefore, in theory, NGS can result in higher polymorphisms of STR loci than CE-STR^6^. Regardless of the sequencing platform adopted, library preparation is a very important step in NGS. First, it requires high-quality extracted genomic DNA or samples on FTA cards. The whole preparation process takes nearly 2 days^7–9^. This is very inefficient and limits the type of sample that can be used.

In this work, we used a 16-plex STR typing system (SeqType^®^R16 Kit) to complete direct library preparation without DNA extraction and adaptor ligation. The efficiency of this system was tested on 593 old blood samples. The NGS data were analysed with SeqVision software (IPE Biotechnology Co., Ltd.) and compared with CE-PCR.

## Results

### Performance for population polymorphism study

#### Time efficiency

It took approximately 4 hours for library preparation, including blood direct multiplex PCR (1.5 hours), mixing of the product (15 minutes), single tube purification (2 hours) and quantification (15 minutes). Faster than ForenSeq^TM^ DNA library preparation, which requires 8 hours, including 3 hours 50 minutes for PCR1, 100 min for PCR2, 155 min for library purification and normalization, including blood direct multiplex PCR (1.5 hours), mixing the of product (15 minutes), single tube purification (2 hours) and quantification (15 minutes)^10^.

#### Data balance among each sequencing runs

Four Personal Genome Machine (PGM) sequencing runs were performed, with each run consisting of 150–151 samples and two DNA controls. As shown in Tables 1, 3.6–4.4 M final library reads were obtained in each Ion 318^TM^ chip sequencing run, with qualified test fragment alignments (94–96%). In addition, 94.4–97.7% (3.5–4.3 M) of the final library reads were qualified by a cut-off of more than 60 bp at the first filtering step of read length.

**Table 1:** Data quality of the three sequencing runs.

Chip ID	Sample number	Final reads	TF alignment	Reads passed length filtering^a^	Barcode and primer sorting^b^	Downstream barcode identification^c^	Matched reads	Reads of each sample (mean ± std.dev)	Reads of per locus per sample(mean ± std.dev)	Reads of per locus per sample	Sample% reported more than 15 loci
1	150	4.4 M	95%	4.27 M	4.0 M	3.7 M	3.6 M	24,053 ± 24,318	1,503 ± 1,520	352~4,466	94%
2	151	4.4 M	97%	4.24 M	4.1 M	3.7 M	3.7 M	24,244 ± 26,667	1,515 ± 1,667	381~4,018	95%
3	150	4.3 M	96%	4.08 M	3.9 M	2.5 M	2.4 M	15,712 ± 14,271	982 ± 892	183~2,311	97%
4	150	3.6 M	94%	3.47 M	3.2 M	2.9 M	2.9 M	19,273 ± 18,228	1,205 ± 1,139	283~3,214	93%
Average	150	4.2 M	95.5%	4.02 M	3.8 M	3.2 M	3.1 M	20,826 ± 21,689	1,301 ± 1,355	359~3,503	94.8%

^a^Length filtering was performed by giving up the reads less than 60 bp.

^b^Barcode and primer sorting was processed by perfect matching to barcode (10 bp) and forward primer sequences (19–23 bp).

^c^Downstream barcodes were specifically chosen sequences (5–10 bp) downstream of the short tandem repeat (STR) repeat region. Detection of this downstream barcode may help to obtain reads that cover the entire STR repeat region. Perfect matching was applied in this filtering step.

Then, 93.4–95.6% (3.2–4.1 M) of the length-qualified reads were successfully sorted into different sample libraries and then into different STR libraries. In the last filtering step, 2.5–3.7 M of the previously sorted reads were qualified as reads with complete repeat sequences by identifying a “downstream barcode”^11^. At last 2.4–3.6 M were successfully matched to the pre-constructed reference, and there were no significant data volume deviations among the four sequencing runs.

#### Locus and inter-locus variation

**Figure 1:**
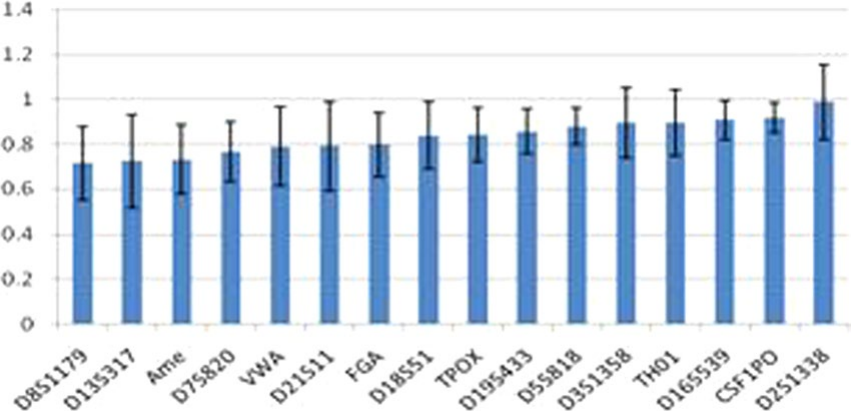
Summary of allele balance of 15 STRs and amelogenin.

**Figure 2:**
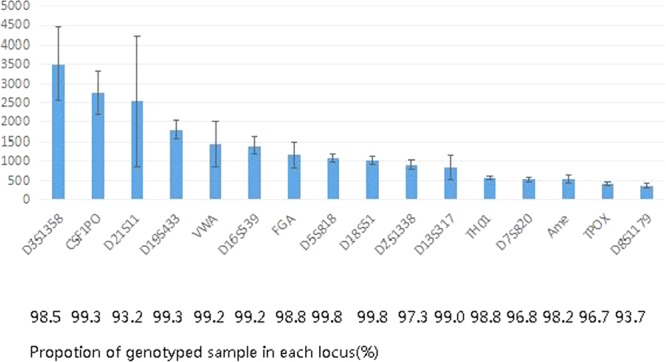
Distribution of read coverage for the 16 loci and the proportion of genotyped samples in each locus.

Table 1 shows that each sample contained 20,826 reads on average, and the coverage ranged from 359 to 3,513 reads on each locus. The performance of inter-locus balance is shown in Fig. 1, and the allele balance ranged from 72–98%. The lowest coverage and allele balance was observed for D8S1179 and TPOX (Fig. 2), indicating that these loci may drop out in poor quality samples. Among all the samples, even the least covered locus, D8S1179, was successfully genotyped in 93.7% of the samples. With the exception of D21S11, the proportion of successfully genotyped samples for the other loci was more than 96.7%. Finally, 94.7% of the samples were successfully genotyped for more than 15 loci.

### Comparison of NGS-STR typing with CE-STR typing

The eight replicates of 2800 M control DNA revealed a complete profile obtaining a 100% correct allele assigning and 100% typing concordance (Supplementary Information 1). The concordance between the length-based CE genotype and the sequence-based NGS genotype was evaluated for all 16 loci in 173 samples (Supplementary Information 2). All but two of the samples showed 100% concordance between NGS-STR typing and CE-STR typing. Table 2 shows that the inconsistencies observed between the two methods were mainly caused by insertion-deletion polymorphisms (indels) in the flanking region. In sample 4591, the D18S51 locus was typed as alleles 15 and 18.1 by CE, but alleles 15 and X by NGS. The X was typed as 18.1 by 54 reads of [AGAA]_14_ A [AAGA][GAGA][GGAA][AGAA]. In sample 4816, D7S820 was typed as alleles 10.1 and 12 by CE, but alleles 10 and 12 by NGS. An inserted T changed the number of downstream homogeneous A’s from nine to ten. Sanger sequencing was used to confirm the results. Sequence alignments are shown in Supplementary Information 3 based on the Forensic STR Sequence Guide^12^. See Sanger sequencing results in Supplementary Information 4.

**Table 2:** Discordance between capillary electrophoresis (CE) and next-generation sequencing (NGS) genotyping methods.

Sample ID	STR	CE	NGS
4591	D18S51	15,18.1	15(61), x(74)
4816	D7S820	10.1,12	10(50), 12(63)

The inconsistent allele call is shown in bold italics. The number of reads for each allele by NGS typing is listed in parentheses.

### Genotyping results and population genetic analyses

Compared with the length-based CE method, the sequence-based NGS method can be used to identify sub-repeat variants with the same length, i.e., isoalleles. Based on the guidelines by ISFG^13^ for nomenclature and the ‘Forensic STR Sequence Structure’ file of the STRIDER STR database^12^, sequence details were shown by STR sequence structure and SNPs described by genome coordinates, and compared with human genome assembly GRCh38 (Supplementary Information 1 for STR structure of all samples with coverage and isoallele frequency for 15 STRs obtained by NGS in Supplementary Information 5). The increase in the number of alleles was due to either a different repeat structure or the presence of single nucleotide polymorphisms (SNPs) in the STR core repeat region and flanking regions. Different repeat structures generated isoalleles with different repeat structures, especially for the seven compound STR loci D2S1338, D21S11, D3S1358, D8S1179, vWA, FGA, and D19S433 in this study. As shown in Table 3, when only the repeat region was considered, an increase in the number of alleles was observed in all seven compound STR loci. D2S1338, D21S11, vWA, D8S1179, and D3S1358 had an increase rate of more than or almost 100% (242, 175, 120, 110, and 88.9%, respectively). However, the rate increased only by 10.5% and 7.1% in FGA and D19S433, respectively. SNPs in repeat regions were also found in simple STRs. For example, a T to C substitution was found in the first repeat of CSF1PO allele 11, and the number of alleles increased from 10 to 11 by NGS typing (Table 3).

**Table 3:** The number of alleles obtained by capillary electrophoresis-based (CE) short tandem repeat (STR) genotyping compared to next-generation sequencing (NGS)-STR genotyping.

Locus	Repeat type	Alleles number identified by CE	Alleles number identified by NGS	Increase rate
D2S1338	Compound	12	41	241.7%
D21S11	Compound	16	44	175.0%
vWA	Compound	10	22	120.0%
D8S1179	Compound	10	21	110.0%
D3S1358	Compound	9	17	88.9%
FGA	Compound	19	21	10.5%
D19S433	Compound	14	15	7.1%
D13S317	Simple	8	23	187.5%
D5S818	Simple	9	19	111.1%
D7S820	Simple	9	16	77.8%
D16S539	Simple	8	14	75.0%
CSF1PO	Simple	10	11	10.0%
D18S51	Simple	17	17	0.0%
TH01	Simple	6	6	0.0%
TPOX	Simple	7	7	0.0%

With the exception of the identification of different STR repeat structures, SNPs in the flanking regions were found in four of the simple STRs, D13S317, D16S539, D5S818, and D7S820, increasing the number of alleles from 75% to 187.5%. In D13S317, for example, a substitution from A to T was frequently found on the first and fifth positions downstream of the TATC repeat. We defined this type of SNP according to the ISFG guidelines (2016)^13^ and analysed the frequency (Supplementary Information 5). Using the sequencing method, the number of alleles did not increase in three simple STR loci, D18S51, TH01, and TPOX (Table 3).

The discrimination power (DP) was calculated to determine the impact of the increase in the number of alleles from NGS typing (Table 4). For loci with a significant increase in the number of alleles, the DP values increased accordingly. For example, the DP increased from 0.801 to 0.862 in D3S1358, from 0.910 to 0.975 in D21S11, and from 0.949 to 0.973 in D2S1338, which is consistent with a high degree of sequence variation as observed in other studies^14–16^. For loci at which the allele number increased due to the presence of SNPs in the flanking regions, an increase in the DP was also observed (e.g., from 0.921 to 0.957 in D13S317). Furthermore, the combined matching probability (CMP) decreased from 1.7 × 10^−15^ to 1.1 × 10^−17^.

**Table 4:** Forensic parameters of 15 STRs in the Chinese Han population obtained by sequence compared to length.

STR	NGS		CE	
	DP	Hexp	DP	Hexp
D2S1338	0.973	0.890	0.949	0.849
D3S1358	0.862	0.760	0.801	0.711
D8S1179	0.975	0.893	0.938	0.833
VWA	0.925	0.816	0.912	0.802
D21S11	0.975	0.897	0.910	0.806
D19S433	0.933	0.829	0.931	0.827
FGA	0.957	0.861	0.957	0.861
D13S317	0.957	0.862	0.921	0.812
D5S818	0.932	0.828	0.870	0.764
D16S539	0.934	0.831	0.891	0.781
D7S820	0.901	0.795	0.882	0.774
CSFIPO	0.840	0.746	0.840	0.746
D18S51	0.943	0.843	0.943	0.843
TH01	0.679	0.648	0.679	0.648
TPOX	0.609	0.612	0.609	0.612

### Mixture studies

The performance of NGS in mixture detection was evaluated with statistical analysis of uniquely identified alleles between two contributors. In total, 15225 data set combinations were analysed among 138 male and 37 female samples, including 9453 male/male sets, 666 female/female sets, and 5106 male/female sets. The alleles that did not overlap with another allele in the mixture or with the n-1 stutter of another allele were defined as uniquely identified alleles. In total, 145599, 10049, and 78097 alleles were not overlapping or stutter reads for the combinations of M/M, F/F, and F/M, respectively, while the numbers were 114886, 8074, and 61921 for CE methods. Uniquely identified alleles increased 28.7 ± 18.7%, 26.3 ± 17.6%, and 28.1 ± 18.9% by sequencing compared to CE methods (Supplementary Information 6).

The effect of sequence variation was indicated by actual mixture sequencing (Supplementary Information 7). For example, in the D2S1338 locus for mixtures of 4575 and 4841, both samples had the allele of 23, but the sequence was [GGAA]_2_[GGAC][GGAA]_13_[GGCA]_7_ in 4575 and [GGAA]_2_[GGAC][GGAA]_14_[GGCA]_6_ in 4841.

Both 4511 and 4539 had the 12 allele in D5S818, and the sequence was [ATCT]_12_ (123775552-A) in 4539 and [ATCT]_12_ in 4511. The 10 allele was [ATCT]_10_ in 4511, while [ATCT]_11_(123775552-A) for the 11 allele in 4539, excluding the possibility of stutter. The additional sequence variation would contribute to the detection of the presence of a DNA mixture.

## Discussion

Compared with the CE method, the NGS strategy is still costly and time consuming. In this study, an Ion platform-based SeqType^®^R system was used. We found that the SeqType^®^R system was comparatively fast for library preparation. The efficiency of the library preparation procedure was improved by the use of a fusion primer to eliminate adaptor ligation and secondary PCR; in addition, separate purification was performed by a single tube operation. These steps are usually required in other NGS library preparation protocols^17^. The whole library preparation process took 4 h, and the data were obtained within 1 day.

Using this highly effective library preparation method, we analysed 593 blood samples that were more than 2 years old and found a large number of isoalleles in 12 STR loci. Eight of these loci had isoalleles due to the presence of polymorphisms in the repeat region, and four of these loci had isoalleles due to the presence of SNPs in the flanking region. Our finding that the number of repeat structures tended to increase is consistent with the published literature^9,18–20^. The impact of NGS-STR typing was reflected by an increase in the DP in each of the 12 STR loci and a decrease in the CMP from 1.7 × 10^−15^ to 1.1 × 10^−17^.

In this study, 94.7% of the samples were successfully genotyped at more than 15 loci using a fixed volume of blood. The report rate would increase from 96% to 99.6% by using uniform DNA as a template (unpublished data from ten sequencing runs, 64 samples each run).

Locus imbalance is one of the challenges in the NGS approach, and loci with lower coverage may drop out in poor quality samples. The most likely reason for the observed bias is amplification imbalance. Further fine-tuning of the primer concentrations should be performed; however, absolute balance is not necessary because less coverage is supposed to be sufficient for STR loci with simple repeat structures.

As shown in this study, direct PCR from blood samples and equal product pooling by volume is particularly suitable for samples with simple and stable characteristics, such as blood cards. Using our study samples, we tried to apply the same panel by reducing the sample throughout each run while adjusting the DNA template volume and product pooling volume based on the DNA source. These data will be released later after performance verification of the SeqType^®^R16 Casework Kit (IPE Biotechnology Co., Ltd.) is conducted.

Compared with the CE method, sequencing affects the allele balance and data quality. Software with an integrated pipeline was used in this study. To improve the efficiency and accuracy, the similarity algorithm and determination flow of SeqVision software were designed as described below.

The most important aspect is to ensure the accuracy of genotyping; therefore, the software was built using a reference database from a large Chinese population to guarantee the accuracy and effectiveness of alignment. Several parameters were provided for the STR call, and the threshold values of parameters were adjusted accordingly for different STR loci. The cut-offs for these parameters were determined based on the results of software training, and the most important criterion for judging the applicability of the threshold was the genotype accuracy compared with the results of the CE profiling method.

Sequence variation would improve the discrimination power of STR, including mixture designation when the contributors had the same genotype by the CE method. In this paper, uniquely identified alleles increased 26.4% by MPS. However, these results must be reported using a new nomenclature, and we reported our result based on the STR sequence nomenclature guideline of the ISFG^13^. We propose reporting sequence variation in several parts, including both length-based results and repeat structures, to maintain compatibility with the current CODIS system and to simplify the nomenclature of indels in the flanking region. In this study, we found an insert of 10 GATAs in the flanking region in D7S820, which is double the number of 10.1 and 10 representing the length-based result and repeat number, respectively. The combination of the two numbers would be a reminder of the insert of the flanking region.

The population data obtained in this study were consistent with a previous study by Gettings *et al*.^7^ that investigated 182 population samples, including African American, Caucasian and Hispanic populations. More than double the number of alleles was detected by sequencing in D2S1338, D21S11, D8S1179, vWA, and D3S1358. Gelardi *et al*.^14^ also reported an increase in allele number for D3S1358 and D21S11 in Danish individuals. In the study by Scheible *et al*.^16^ for a U.S. population, allele counts did not increase in D2S1338 by sequencing method. In our study, the allele number increased by 242% in D2S1338. Differences between the findings of different studies might be caused by sample size and population resources.

## Materials and Methods

All samples were collected from the anonymous donors who gave their permission for DNA analysis and scientific publication. Written informed consent was obtained for each participant. This study was approved by the Ethics Committee of Sun Yat-sen University (permit number: 2017-040). All the methods were carried out in accordance with the approved guidelines of the Academy of Forensic Sciences, Ministry of Justice, P.R. China.

### Population samples

A total of 593 samples from unrelated Chinese Han individuals were sequenced and analysed in this study. One- to three-year-old blood samples stored at −20 °C were used as the PCR template. Four PGM sequencing runs were performed with each run consisting of 150–151 samples and two 2800 M controls (Promega Corporation, Madison WI, USA). All samples were collected with written consent from the anonymous donors who gave their permission for DNA analyses and scientific publication. Nine blood mixtures were prepared from two males (4511/4539), two females (4575&4851), and male and female (4533&4851) at 3 ratios (1:9, 1:1, 9:1).

### NGS library preparation

Libraries containing 16 STR amplicons (D3S1358, D5S818, D7S820, D8S1179, D13S317, D16S539, D18S51, D21S11, CSF1PO, FGA, TH01, TPOX, vWA, D2S1338, D19S433, and amelogenin) attached by adaptors were directly amplified from whole blood using the fusion primer pool and blood direct DNA polymerase (SeqType^®^R16 Kit, IPE Biotechnology, Beijing, China). The details of the fusion primer have been reported previously^11^.

PCR was performed in a 10 µL reaction volume as described previously^11^ with 1 µL of whole blood as the template. Equal volumes of 192–204 PCR products were pooled (190–202 samples and two 2800 M controls), and then 50 µL of this pool was purified with 1.2 × volume SeqType^®^R16 DNA clean-up reagent (IPE Biotechnology, Beijing, China). PCR products of nine blood mixtures were pooled together and purified in the same way.

The purified sequencing pool was quantified using a Qubit^®^ dsDNA HS Assay Kit (Thermo Fisher Scientific, Waltham, MA, USA) and diluted to 0.016 ng/µL based on the quantification results. A total of 0.4 ng of product (in 25 µL) was used as the library for NGS detection. Emulsion PCR and subsequent positive bead enrichment were performed on the Ion Chef instrument (Thermo Fisher Scientific), and sequencing was performed using the Ion PGM system (Thermo Fisher Scientific) on the Ion 318^TM^ chip (Thermo Fisher Scientific) according to the instructions of the Ion PGM Hi-Q Chef Kit.

### Data processing and genotype calling

The in-house software SeqVision V1.5^21,22^, which was based on sequence alignment, was designed to perform STR genotype calling. Sequence alignment was mainly based on the Needleman-Wunsch algorithm; as an example of dynamic programming, the discipline was invented by Richard E. Bellman in 1966^23^. The reference bank in this software was based on Chinese Han population data collected by IPE Biotechnology Co., Ltd. The analysis was performed similar to the pipelines in a previous study^11,24^. Allele (30× to 50×) or locus (80×) coverage cut-offs were set up by assuming a binomial distribution of heterozygous allele coverage, based on absolute amplification balance and no read length effect on sequencing quality. The adjustable parameters were described in detail in a previous study^24^.

### Genotyping by CE-STR methods

A total of 173 samples were also genotyped using the GlobalFiler^TM^ Express Kit (Applied Biosystems, Inc., Foster City, CA, USA), which co-amplified the same 16 loci in this study. The PCR products were genotyped with capillary electrophoresis on a 3130xl (Applied Biosystems, Inc.). Genotyping was carried out using GeneMapper^®^ID-X1.4. The genotyping results obtained by NGS-STR and CE-STR methods were compared one by one.

## Supplementary information

Supplementary information 1

Supplementary information 2

Supplementary information 3

Supplementary information 4

Supplementary information 5

Supplementary information 6

Supplementary information 7

## Data Availability

All data included in this study are available upon request by contact with the corresponding author. (Wenshen Dai, +86 17090146703, vensondai@163.com).
